# A novel quadruplex real-time PCR method for simultaneous detection of *Cry2Ae* and two genetically modified cotton events (GHB119 and T304-40)

**DOI:** 10.1186/1472-6750-14-43

**Published:** 2014-05-16

**Authors:** Xiang Li, Xiuxiu Wang, Jielin Yang, Yueming Liu, Yuping He, Liangwen Pan

**Affiliations:** 1 GMO Detection Laboratory, Shanghai Entry-Exit Inspection and Quarantine Bureau, 1208 Minsheng Road, Shanghai 200135, PR China; 2 Institute of Biotechnology, East China University of Science and Technology, 130 Meilong Road, Shanghai 200237, PR China

**Keywords:** *Cry2Ae*, Event-specific, GHB119, Quadruplex real-time PCR, T304-40

## Abstract

**Background:**

To date, over 150 genetically modified (GM) crops are widely cultivated. To comply with regulations developed for genetically modified organisms (GMOs), including labeling policies, many detection methods for GMO identification and quantification have been developed.

**Results:**

To detect the entrance and exit of unauthorized GM crop events in China, we developed a novel quadruplex real-time PCR method for simultaneous detection and quantification of GM cotton events GHB119 and T304-40 in cotton-derived products (based on the 5′-flanking sequence) and the insect-resistance gene *Cry2Ae*. The limit of detection was 10 copies for GHB119 and *Cry2Ae* and 25 copies for T304-40. The limit of quantification was 25 copies for GHB119 and *Cry2Ae* and 50 copies for T304-40. Moreover, low bias and acceptable standard deviation and relative standard deviation values were obtained in quantification analysis of six blind samples containing different GHB119 and T304-40 ingredients.

**Conclusions:**

The developed quadruplex quantitative method could be used for quantitative detection of two GM cotton events (GHB119 and T304-40) and *Cry2Ae* gene ingredient in cotton derived products.

## Background

Genetically modified (GM) crops have been commercially cultivated for over 17 years and the global planting area has increased 100-fold [1]. Along with the rapid development of GM organisms (GMO), food safety and environmental safety concerns about GMO increasingly attracted public attention worldwide. To respond to these concerns, a series of laws and regulations have been issued by many countries throughout the world, highlighting labeling policies for GMO-derived products to protect individual consumer’s “right to know”. To ensure the implementation of GMO labeling regulations, it is necessary to develop appropriate, rapid qualitative and quantitative methods for GMO detection.

Nucleic acid-based and protein-based techniques are two widely used methods for GMO detection. Due to their high specificity, sensitivity, operability and repeatability, nucleic acid-based methods (such as PCR, real-time PCR, isothermal amplification techniques and others) are widely employed for the detection of GMO events [2,3]. As the quantity of exogenous genes and GM crop events increase, several multiplex methods for simultaneous detection of more than one genetic element have been developed, such as multiplex PCR, microarray analysis, suspension array analysis, microdroplet-based PCR and others [4-6]. These techniques are especially useful for detecting GM events in complex or mixed samples that contain multiple exogenous insertions. Due to the limited stability and repeatability of microarray and suspension array-based PCR methods, such methods are unsuitable for routine analysis of GM events in imported and exported goods. Therefore, most methods based on multiplex techniques involve conventional multiplex PCR, such as a six-fold screening method for detecting 35S, pNOS, tNOS, NPTII, CP4-EPSPS and PAT [7], methods for simultaneously detecting Cry1Ac, Cry2Ab, pCaMV35S, tNOS, NPTII, aadA, uidA, and MON15985 event [8] and multiplex PCR analysis for detection of four GM maize, five GM soybean and six GM canola elements, among others [9]. Compared with conventional PCR, real-time PCR has a higher sensitivity and a shorter detection time and is easier to perform. However, developing a multiplex real-time PCR system is proved to be difficult due to serious mutual interference between pairs of primers and probes that greatly reduces the detection sensitivity. Currently, only a few real-time multiplex PCR methods are reported and maximum four targets are simultaneously detected [10,11].

Cotton is an economically important crop with a global planting area of approximately 36 million hectares in 2012, 81% of which was planted with more than 30 GM cottons [1]. GM cotton is planted in 13 countries and six GM cottons, MON531, MON1445, MON15985, LLcotton25, MON88913 and GHB614, have been certificated for food and feed production in China since 2004 [12]. GHB119 and T304-40 are developed by Bayer Crop Science (Monheim, Germany) with both insect- resistance and herbicide-tolerance characteristics. GHB119 contains two exogenous genes (*Cry2Ae* and *bar*) while T304-40 contains two exogenous genes (*Cry1Ab* and *bar*). To date, GHB119 and T304-40 have been approved for cultivation in Japan and the United States and for food or feed processing in Canada, the United States, Australia and New Zealand [13]. However, to date, no detection methods for these two GM cotton events have been reported, which is also true for the *Cry2Ae* gene.

In this study, we analyzed the *Cry2Ae* gene sequence and the 5′-flanking sequence of GM cotton events GHB119 and T304-40. We then developed three detection systems based on a specific fragment of *Cry2Ae* and 5′ event-specific sequences of GHB119 and T304-40. A quadruplex quantitative real-time PCR method together with the cotton endogenous gene *ACP1* detection system was established for simultaneous detection and quantification of GHB119, T304-40 and *Cry2Ae* in cotton-derived products. Due to the high homology between *Cry2Ae* and other *Cry* genes, to improve the specificity and sensitivity of this system for *Cry2Ae* detection, locked nucleic acids (LNA) were included in the *Cry2Ae* probe.

## Methods

### Sample collection and preparation

Certified reference materials (CRMs) of GM cotton (*Gossypium*) GHB119 (ERM- BF428c), T304-40 (ERM-BF429c) and 281-24-236 × 3006-210-23 (ERM-BF422d), GM maize (*Zea mays* L*.*) Bt11 (ERM-BF412f), MON98140 (ERM-BF427d), NK603 (ERM-BF415f) and MON810 (ERM-BF413k) and GM soybean (*Glycine max*) GTS40-3-2 (ERM-BF410k), DP305423 (ERM-BF426d) and DP356043 (ERM- BF425d) were purchased from the Institute for Reference Materials and Measurements (IRMM), Joint Research Center (JRC) of European Commission (Geel, Belgium). Some GM materials, such as GM cotton MON88913 (0906-D), MON531 (0804-C), MON15985 (0804-D) and MON1445 (0804-B) and GM soybean MON89788 (0906-B) were purchased from the American Oil Chemists’ Society (AOCS) (Urbana, IL, USA). Dry seed powders of GM rice (*Oryza sativa*) TT51-1, GM canola (*Brassica campestris L.*) T45, GM maize CBH351, GM cotton GK19 and SGK321 and a conventional cotton variety were provided by corresponding developers.

To verify the sensitivity of the established quadruplex real-time PCR method, six different percentages of mixed-cotton samples were produced using a Freezer Mill (Spex SamplePrep, Metuchen, NJ, USA). First, 10.0% CRM of GM cotton GHB119 powder (0.60 g) was mixed with non-GM cotton powder (0.60 g), resulting in a 5.0% (w/w) sample (labeled A1). Next, 0.60 g of A1 was mixed with 0.90 g of non-GM cotton powder, resulting in a 2.0% GHB119 sample (labeled A2). Then, a 1.0% GHB119 sample (labeled A3) was obtained by mixing 0.60 g of A2 with 0.60 g of non-GM dry cotton powder. Similarly, samples containing 5.0% (B1), 2.0% (B2) and 1.0% (B3; w/w) T304-40 powder were also prepared using the same mixing pattern. Then, six blind samples (S1-S6) containing both GHB119 and T304-40 powder were prepared. The mixing method and GM content of six samples were listed in Table 1.

**Table 1:** Mixing method and GM content of six prepared blind samples

**Sample name**	**GM content (%) (w/w)**		**Mixing method**
	**GHB119**	**T304-40**	
S1	0.5	5.0	0.2 g A3* + 0.2 g of 10.0% CRM of T304-40
S2	5.0	1.0	0.2 g of 10.0% CRM of GHB119 + 0.2 g of B2*
S3	2.5	0.5	0.2 g of A1* + 0.2 g of B3*
S4	1.0	2.5	0.2 g of A2* + 0.2 g of B1*
S5	0	0.5	0.2 g of non-GM cotton + 0.2 g of B3*
S6	0.5	0	0.2 g of A3* + 0.2 g of non-GM cotton

*A1: 5.0% (w/w) GM cotton GHB119 sample; A2: 2.0% (w/w) GM cotton GHB119 sample; A3: 1.0% (w/w) GM cotton GHB119 sample; B1: 5.0% (w/w) GM cotton T304-40 sample; B2: 2.0% (w/w) GM cotton T304-40 sample; B3: 1.0% (w/w) GM cotton T304-40 sample.

### DNA extraction

Genomic DNA samples were isolated and purified from approximately 200 mg dry cotton seed powder using a Plant Genomic DNA Extraction Kit (Tiangen Biotech Co., Ltd., Beijing, China) according to the manufacturer’s instructions. The concentrations and quality of the extracted DNA samples were measured with a Nanovue Plus Spectrophotometer (GE Healthcare Bio-sciences, PA, USA) at 260, 280, 230 and 320 nm and evaluated by examining the A_260_/A_280_ and A_260_/A_230_ ratios, followed by electrophoresis on 0.8% agarose gels. All DNA solutions were stored at -20°C. In addition, the genomic DNA copy numbers were calculated by referring to the haploid genome size of cotton.

### Design of primers and probes

After analyzing the sequences of *Cry2Ae* (Accession number: AX513526.1) and other Bt genes, such as *Cry2Ab*, *Cry1Ab*, *Cry1Ac*, *Cry3A* and *Cry9C* (Accession numbers are AB702969.1, KF303141.1, KF630361.1, X70979.1 and AY346129.1, respectively) by alignment using DNAMAN 6.0.40 software (Lynnon Biosoft Corp., Quebec, Canada) [14], a fragment of the *Cry2Ae* gene with minimum sequence similarity with the other *Cry* gene was chosen for the design of primers and probes. To improve the specificity of the probe for amplifying *Cry2Ae*, two nucleic acids on the probe were replaced by LNA (Table 2). The 5′-specific flanking sequences of the GM cotton events GHB119 and T304-40 (http://www.ncbi.nlm.nih.gov/nucleotide/) were analyzed, and primer pairs GHB119-F1/R2 and T304-F1/R2 and probes GHB119-P and T304-P were designed using Primer Express 3.0 software (Applied Biosystems, Foster City, CA, USA). The primer pair and probe 18SrRNA-F/R/P [15] that is specific to the *18SrRNA* gene of eukaryotes were used as an internal control. Primer pair ACP-FP/RP and probe ACP-P were designed to detect the endogenous *ACP1* gene (Accession number: U48777.1) of cotton. Four fluorescent channels were employed, using the fluorescent reporter dyes FAM, HEX, ROX and CY5 to label the 5′-ends of the probes GHB119-P, T304-P, ACP-P and CRY2E-P, respectively. Correspondingly, the quencher dyes BHQ1 (for FAM and HEX), BHQ2 (for ROX) and BHQ3 (for CY5) were used to label the 3′-ends of the probes, respectively. FAM and BHQ1 dyes were used to label 18SrRNA-P. The sequences of the primers and probes used in this study are listed in Table 2. The primers and fluorescence-labeled probes were synthesized and purified by Shanghai Huirui Biotechnology Co., Ltd. (Shanghai, China).

**Table 2:** Primers and probes used for quadruplex real-time PCR assays in this study

**Target**	**Purpose**	**Primer/probe name**	**Sequence (5′-3′)**	**Amplicon length (bp)**	**Reference**
18S rRNA	Eukaryotes gene	18SrRNA-F	CCTGAGAAACGGCTACCAT	137	19
		18SrRNA-R	CGTGTCAGGATTGGGTAAT		
		18SrRNA-P	FAM-TGCGCGCCTGCTGCCTTCCT –BHQ1		
ACP1	Cotton endogenous gene	ACP-FP	ATGAACCAGGGAAGAAGCACC	97	This study
		ACP-RP	CCTTATCCACGGTCTCTTGTTTG		
		ACP-P	ROX-CATTTACGATGCGTCCAATGCCTG-BHQ2		
GHB119	Event-specific detection	GHB119-F1	AAAACTTTGTGCAGCCTTCG	130	This study
		GHB119-R2	CGCAAACTAGGATAAATTATCGC		
		GHB119-P	FAM-TCCCCCTATCTTGCTAAATGGCTCC-BHQ1		
T304-40	Event-specific detection	T304-F1	GTCATTGTAGGGAGTTTGTCCAA	118	This study
		T304-R2	CTGTAGCCACAACACCACTTTG		
		T304-P	HEX-TTAATCCCAGTACTCGGCCGTC-BHQ1		
Cry2Ae	Gene-specific detection	CRY2E-F1	CTTGCTCTACTTTCCTTCCTCC	114	This study
		CRY2E-R2	CGAAAGACTCAGTTTGCCAGT		
		CRY2E-P	CY5-CAAGCCAAGA + C + CTAACGAAAGGAG-BHQ3*		

*The base before symbol “+” was decorated with LNA.

### Real-time PCR conditions

Quadruplex real-time PCR was performed in an Applied Biosystems ViiA 7 Real-Time PCR System (Applied Biosystems, Foster City, CA, USA) in a 25 μl reaction volume. Each reaction included 12.5 μl of 2× HR qPCR Master Mix I (Shanghai HuiRui Biotechnology Co., Ltd, China), 5 μl of plant DNA templates and 200 nM of primer ACP-FP/RP with 100 nM of ACP-P probe and 240 nM each of primer GHB119-F1/R2, T304-F1/R2 or CRY2E-F1/R2 with 120 nM each of GHB119-P, T304-P or CRY2E-P probe. Real-time PCR amplification was carried out with the following program: 95°C for 10 min followed by 45 cycles of 95°C for 15 s and 60°C for 1 min. The fluorescence was monitored during each PCR cycle at the annealing and extension step (60°C).

### Specificity test

To determine the specificity of the three established real-time PCR assays for GM cotton GHB119, T304-40 and *Cry2Ae*, three primer pairs with the corresponding probe were tested by amplifying the genomic DNA samples of relevant GM plant events, including 10 GM cotton events (GHB119, T304-40, 281-24-236, 3006-210-23, MON88913, MON531, MON15985, MON1445, GK19 and SGK321), five GM maize events (Bt11, MON98140, NK603, CBH351 and MON810), four GM soybeans (GTS40-3-2, 305423, 356043 and MON89788), one GM rice (TT51-1), one GM canola (T45) and a conventional cotton variety (ZM-1).

### Detection limit assay

To test the limit of detection (LOD) and quantification (LOQ) of the established quadruplex real-time PCR method, four gradient dilutions of DNA template were prepared. To obtain the DNA solution containing both GHB119 and T304-40 events, the DNA samples of 10% GHB119 (W/W) and 10% T304-40 (W/W) were mixed firstly. The same concentration (100 ng/μl) and equal volumes GM cotton GHB119 and T304-40 DNA solutions were mixed thoroughly, yielding the “first mixture” of 100 ng/μl DNA sample with 5 ng/μl GHB119 and 5 ng/μl T304-40 content. According to the mean molecular weight and size of the cotton genome, 1 ng cotton DNA equals approximately 464 copies of DNA. In the “first mixture”, the concentration of the cotton haploid genomic DNA was 4,640,000 copies/μl, and those of GHB119 and T304-40 were 2320 copies/μl, respectively. The “first mixture” was serially diluted in 0.1 × TE buffer (1 mM Tris–HCl, 0.1 mM EDTA ⋅ Na, PH 8.0) to final concentrations of 200, 100, 40 and 20 copies/μl of total cotton haploid genomic DNA, which contained 10, 5, 2 and 1 copies/μl for both GHB119 and T304-40 content, respectively, because that the contents of GM cotton GHB119 and T304-40 were both 5% (W/W) in the “first mixture”. Each sample was amplified in five parallel reactions, and the entire experiment was repeated four times. The absolute LOD and LOQ values represent the lowest amount or concentration of initial template DNA that could be reliably detected and quantified with a ≥95% confidence interval [16]. The LOD value was determined and validated by calculating the number of positive results in 20 reactions.

### Construction of standard curves

To quantify GHB119 and T304-40 contents in the blind samples, four standard curves were first constructed through four fluorescent channels in one round of amplification. DNA solutions were prepared by serially diluting the prepared “first mixture” using 0.1 × TE, which contained 5 ng/μl GHB119 and 5 ng/μl T304-40. Six concentrations of cotton DNA, including 100, 20, 4, 2, 0.8 and 0.4 ng/μl, which equal approximately 46,400, 9,280, 1,856, 928, 371 and 185 copies/μl of haploid cotton genomic DNA, were employed for amplification using the quadruplex and simplex PCR assays. According to the content of GHB119 or T304-40, which was 5.0% in the above six concentrations, the number of copies was approximately 2,320, 464, 92.8, 46.4, 18.6 and 9.3 per μl for GHB119 or T304-40 content. In this assay, four parallel reactions were performed for each concentration, and the entire experiment was repeated three times. The Ct values that deviated considerably from the average value were rejected. Eight standard curves (four by quadruplex and four by simplex) were plotted using Ct values from six concentrations of genomic DNA against the logarithm of the number of copies of DNA. Furthermore, the constructed standard curves were evaluated by amplification efficiency (E = [10^(-1/slope)^ -1] × 100%) and square regression coefficient (R^2^), respectively. The repeatability of four parallel reactions in one round and three experiments was validated by examining the standard deviation (SD) and relative standard deviation (RSD).

### Analyses of blind samples using the quadruplex real-time PCR method

To evaluate the quadruplex PCR method for GM cotton GHB119, T304-40 and *Cry2Ae* quantification, six blind samples were prepared, including S1 (containing 0.5% GHB119 and 5.0% T304-40), S2 (5.0% GHB119 and 1.0% T304-40), S3 (2.5% GHB119 and 0.5% T304-40), S4 (1.0% GHB119 and 2.5% T304-40), S5 (0.5% GHB119 and 0% T304-40) and S6 (0% GHB119 and 0.5% T304-40). Four replicates were carried out per sample. The precision and accuracy of the quadruplex quantitative real-time PCR method were verified by examining the bias, SD and RSD values.

## Results and discussion

### Development of the quadruplex real-time PCR method for GM cotton GHB119, T304-40 and Cry2Ae detection

**Figure 1:**
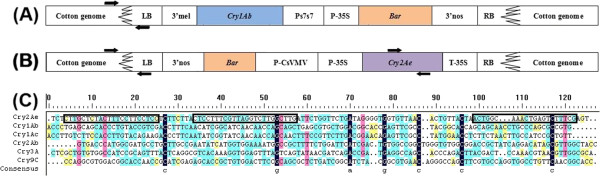
**Schematic diagram of exogenous insertion of GM cotton events T304-40 and GHB119 and alignment of six Cry protein gene sequences. (A–B)** Schematic diagram of exogenous insertion of GM cotton events T304-40 **(A)** and GHB119 **(B)**. LB: left border sequence of T-DNA; 3′mel: terminator from *Flaveria bidentis*; Cry1Ab: gene encoding Bt endotoxin of *Bacillus thuringiensis*; Ps7s7: duplicated promoter from subterranean clover stunt virus; P-35S: 35S promoter from cauliflower mosaic virus; Bar: sequence encoding the PAT (phosphinothricin acetyl-transferase) enzyme of *Streptomyces hygroscopicus*; 3′nos: 3′-terminator of nopaline synthase gene from *Agrobacterium tumefaciens*; Cry2Ae: gene encoding a Bt endotoxin of *Bacillus thuringiensis*; T-35S: 35S terminator from cauliflower mosaic virus; P-CsVMV: promoter derived from Cassava vein mosaic virus; RB: right border of transposon from *Agrobacterium tumefaciens*. Arrows show the primers location developed in this study. **(C)** DNA fragment sequences of six Cry protein genes showing the locations of the designed primers and probes.

High-throughput, rapid detection methods have attracted increasing attention due to their considerable advantages. In this study, to rapidly detect two unapproved GM cotton events in China and their inserted gene, we developed a novel quadruplex quantitative real-time PCR detection method, combined with the LNA technique, for simultaneous detection and quantification of GM cotton events GHB119 and T304-40 as well as *Cry2Ae*. Primer pairs and probes for GHB119 and T304-40 detection were designed based on both 5′-flanking sequences of the two events (Figure 1). Nearly 10 Cry proteins have been used to provide insect-resistance characteristics in GM crops (such as *cry1Ab*, *cry2Ab, cry9C* and so on), and the homology among these *Cry* genes is as much high as 95%. The alignments of five *Cry* genes in GM crops and the specific fragment used to design the primers and probe are shown in Figure 1. To improve the specificity of the detection method, two bases on the probe used for *Cry2Ae* gene detection were modified by decoration with LNA (Table 2).

Since quadruplex real-time PCR employs eight primers and four probes in a single amplification tube, mutual interference and inhibition may occur. Therefore, to obtain an amplification curve with the strongest fluorescent signals and the lowest Ct values, multiple pairs of primers and probes were designed and tested. We also tested four quantitative PCR (qPCR) master-mix solutions and used concentrations of primers ranging from 150 to 300 nM and probes ranging from 75 to 150 nM. We determined that the optimal amplification system comprises 1 × HR qPCR Master Mix I, 240 nM of each primer GHB119-F1/R2, T304-F1/R2 and CRY2E-F1/R2 and 200 nM of the primer pair ACP-FP/RP, 120 nM of each probe GHB119-P, T304-P and CRY2E-P and 100 nM of the probe ACP-P.

### Specificity test of the real-time PCR method

**Figure 2:**
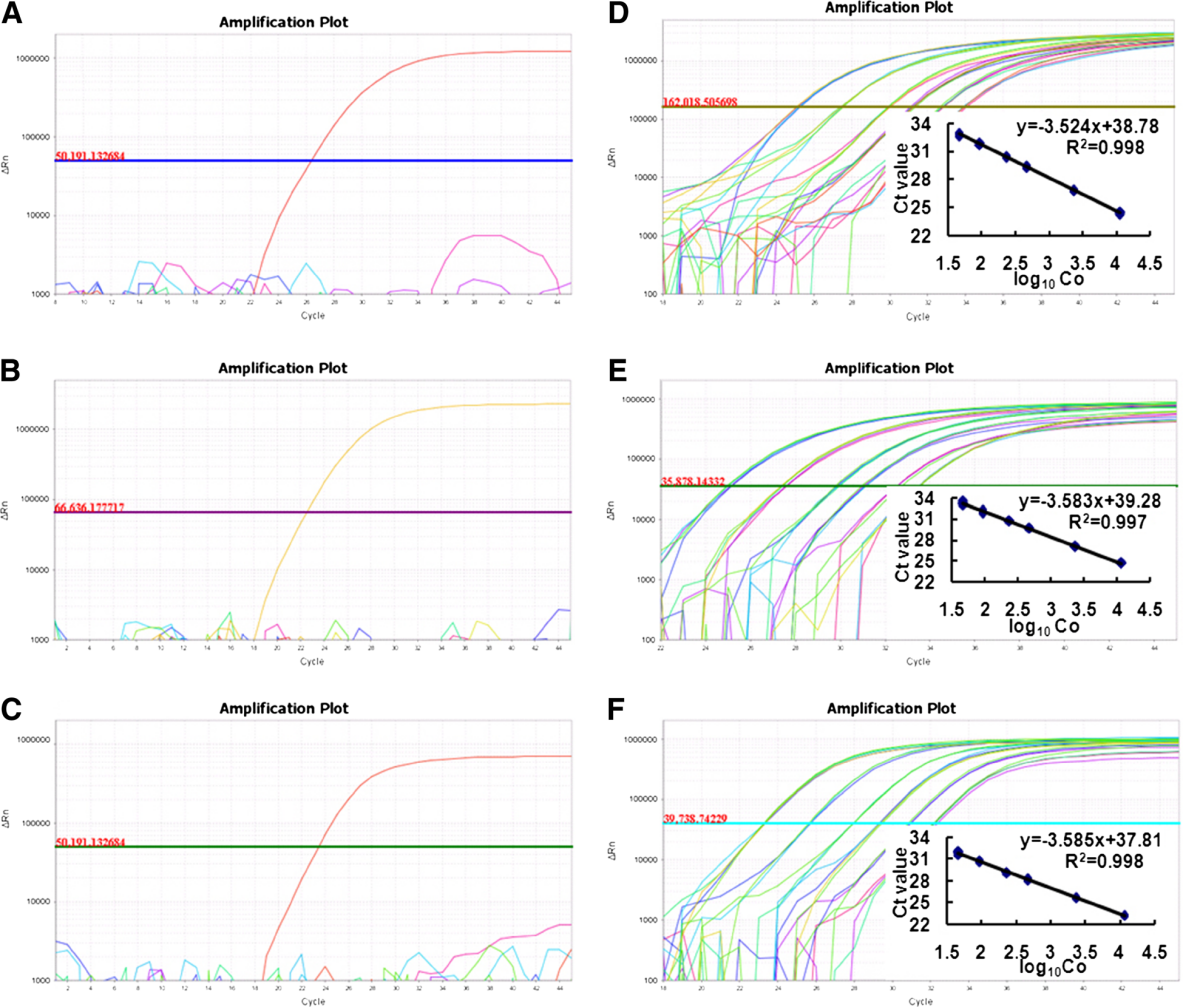
**Specificity tests and standard curves of the real-time PCR method designed to detect GM cotton GHB119, T304-40 and*Cry2Ae*. (A–C)** Specificity tests using 22 plant samples as templates for GHB119 **(A)**, T304-40 **(B)** and *Cry2Ae***(C)** detection method. **(D–F)** Amplification plots and standard curves from six concentrations (2,320, 464, 92.8, 46.4, 18.6 and 9.3 copies/μl) of GHB119 **(D)**, T304-40 **(E)** or *Cry2Ae***(F)** ingredient.

To evaluate the specificity of the designed primer pair/probe for GHB119, T304-40 or *Cry2Ae*, 22 DNA samples from various plants including cottons were amplified. To determine if the extracted DNA samples were suitable for amplification, an *18SrRNA* gene fragment was first amplified, which showed significant fluorescent amplifications in all 22 DNA samples (data not shown). Furthermore, amplification curves of the cotton endogenous gene *ACP1* were observed only from cotton-derived samples (data not shown). The successful amplification of both *18SrRNA* and *ACP1* genes indicated that the extracted 22 DNA samples were suitable for real-time PCR analysis.The specificity test for above mentioned three targets was carried out using simplex real-time PCR. In the test for primer pair/probe GHB119-F1/R2/P for GM cotton GHB119 detection, the amplification curve (with a Ct value of nearly 26) was present only in GHB119 genomic DNA sample (Figure 2A). Similarly, for primer pair/probe T304-F1/R2/P, the amplification curve was only observed in T304-40 genomic DNA sample (Figure 2B). In addition, the expected amplification curve for primer pair/probe CRY2E-F1/R2/P test (with a Ct value of approximately 23) was appeared only in GHB119 genomic DNA sample (Figure 2C). These results indicated that all three designed primer pairs/probes were highly specific and could be used for the quadruplex real-time PCR assay.

### Sensitivity tests

LOD and LOQ are two of the most important parameters used to evaluate a newly established qPCR method. Furthermore, it is also important that the fluorescent signals and Ct values are similar in multiplex and simplex PCR when using the same DNA template.

**Figure 3:**
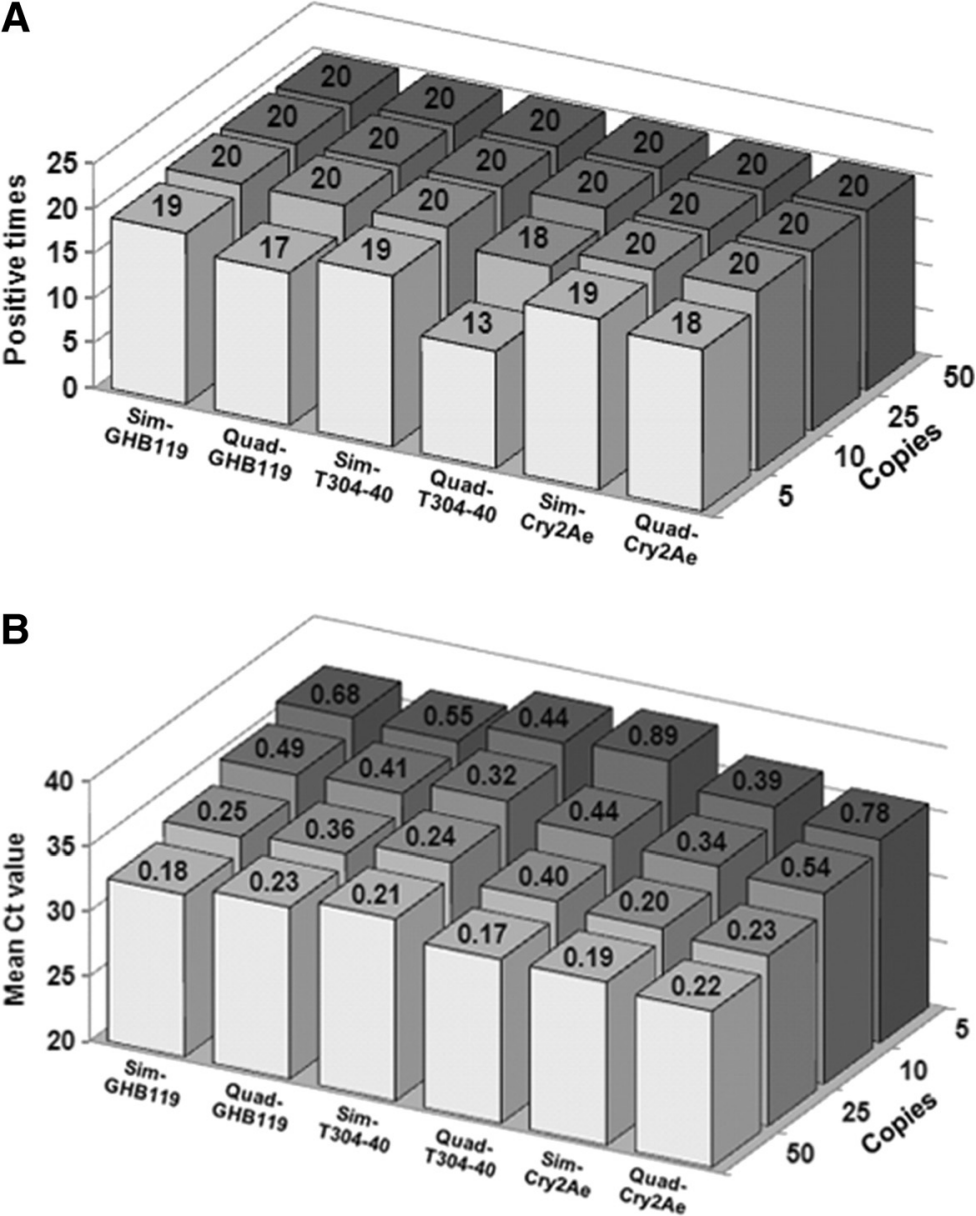
**Results of sensitivity tests for the established real-time PCR method. (A)** Number of positive amplifications out of 20 using four concentrations (10, 5, 2 and 1 copies/μl) of GHB119 or T304-40 ingredient by simplex and quadruplex PCR assays. **(B)** Mean Ct values and SD for GHB119, T304-40 and *Cry2Ae* detection via simplex and quadruplex PCR assays using four concentrations (10, 5, 2 and 1 copies/μl) of GHB119 or T304-40 DNA as template. The number above the columns is standard deviations.

In this study, to determine the LOD and LOQ of our quadruplex real-time PCR method, four concentrations of serially diluted DNA samples, including 10, 5, 2 and 1 copies/μl of GHB119 or T304-40 (the sample with a concentration of 10 copies/μl contained both 10 copies/μl GHB119 and 10 copies/μl T304-40, and so on) were employed. When 10 and 5 copies/μl (with 5 μl templates) of DNA were used as template amplification curves were obtained in all 20 reactions for GHB119, T304-40 and *Cry2Ae* in either quadruplex or simplex PCR system (Figure 3A). When 2 copies/μl (with 5 μl templates) of DNA were used as template, amplification curves were obtained in all 20 reactions for GHB119 and *Cry2Ae* in either quadruplex or simplex PCR. However, when 10 copies of DNA was used as template for the T304-40 test (18 out of 20 reactions were positive in the quadruplex PCR assay while all 20 simplex PCR reactions were positive (Figure 3A). Differences were also observed in amplifications using five copies (5 μl templates) of DNA as template. Positive signals appeared 19 out of 20 reactions for all three targets using simplex PCR and in 17, 13 and 18 out of 20 reactions for GHB119, T304-40 and *Cry2Ae* detection by quadruplex PCR, respectively. In other words, the lowest levels of DNA that could be reliably detected using the quadruplex PCR method were 10, 25 and 10 copies (2, 5 and 2 copies/μl with 5 μl templates, respectively) for GHB119, T304-40 and *Cry2Ae*, respectively. These LOD values were similar to those obtained using simplex PCR. Note that 10 copies of haploid genomic DNA are equal to approximately 0.02 ng of cotton DNA.

We also calculated the mean Ct values using the above four concentrations of genomic DNA as template in both quadruplex and simplex PCR systems. No significant differences were observed between the quadruplex and simplex PCR results for all three targets (Figure 3B). It is gratifying that the Ct values obtained by quadruplex PCR targeting GM cotton T304-40 and *Cry2Ae* from all four concentrations of cotton DNA tested were less than the results obtained using the simplex PCR method. In the amplifications targeting GHB119 event, the Ct values were similar in both quadruplex and simplex PCR systems. These results indicated that little or no interference occurred among the four groups of primers and probes when amplified in a single reaction tube.

To obtain the LOQ of the quadruplex PCR method, we calculated the SD of Ct values from 20 replicates using four concentrations of genomic DNA. The SD values were all <0.25 for GHB119, T304-40 and *Cry2Ae* detection when 50 copies of genomic DNA were used as templates (Figure 3B). For GHB119 and T304-40 quantification, reliable Ct values were observed when ≥25 copies of DNA were used as template in simplex PCR and ≥50 copies of DNA were used in quadruplex PCR. For *Cry2Ae*, the lowest concentration of template that could reliably be used for detection was 25 copies for both simplex and quadruplex PCR. Therefore, the LOQs of the quadruplex real-time PCR method were 50 copies of genomic DNA for GHB119 and T304-40 events and 25 copies for *Cry2Ae*. Thus, 0.44% to 100% GHB119 and T304-40 ingredient in 100 ng mixed samples could be accurately quantified and that value for *Cry2Ae* gene was a 0.22%-100% in 100 ng mixed samples.

### Construction of standard curves

To quantify the GM content in blind samples, after optimizing the reaction conditions, we constructed standard curves using our quadruplex real-time PCR method. Six concentrations of mixed genomic DNA were used, including approximately 2,320, 464, 92.8, 46.4, 18.6 and 9.3 copies/μl for GHB119 or T304-40. Simplex real-time PCR was also carried out under the same conditions. Two parameters, that is, E and R^2^, were employed to evaluate the standard curves. The amplification plots and standard curves were shown in Figure 2. The R^2^ values were all >0.99 (0.993–0.998 range) for both the quadruplex and simplex PCR assays for GHB119, T304-40 and *Cry2Ae*, indicating that the six standard curves had good linearity and relativity (Table 3). The PCR efficiencies were high, ranging from 90 to 95% in both the quadruplex and simplex PCR assays. Specifically, for GM cotton GHB119 detection, PCR efficiencies were 90.4% and 92.2% for simplex and quadruplex PCR, respectively. For GM T304-40, PCR efficiencies were 91.3% and 90.1% for simplex and quadruplex PCR, respectively, and these values for *Cry2Ae* detection, were 92.5% and 90.1% for simplex and quadruplex PCR, respectively (Table 3). The good linearity observed between DNA quantity and fluorescence values (Ct) and the high PCR efficiencies indicated that the quadruplex real-time PCR method would be suitable for practical application in the quantitative detection GM cotton GHB119, T304-40 and *Cry2Ae* in blind samples.

**Table 3:** **Parameters of standard curves constructed using the quadruplex and simplex PCR assays for GM cotton GHB119, T304-40 and** ***Cry2Ae*** **detection**

**Target**		**Linear equation**	**R**^**2**^	**PCR efficiency (%)**
**GHB119**	Simplex	y = -3.575x + 43.541	0.997	90.4
	Quadruplex	y = -3.524x + 38.780	0.998	92.2
**T304-40**	Simplex	y = -3.549x + 42.421	0.993	91.3
	Quadruplex	y = -3.583x + 39.275	0.997	90.1
***Cry2Ae***	Simplex	y = -3.516x + 42.034	0.998	92.5
	Quadruplex	y = -3.585x + 37.809	0.998	90.1

### Repeatability of the quadruplex real-time PCR method

The repeatability of the quadruplex real-time PCR assay for GHB119, T304-40 or *Cry2Ae* was evaluated by examining the SD and RSD values of four parallel replicates (SD^r^ and RSD^r^) and three experiments (SD^R^ and RSD^R^). The results revealed that the SD and RSD values were all in the acceptable range. Specifically, the SD^r^ values were less than 0.19 using six concentrations of DNA as template for detection of GHB119, T304-40 and *Cry2Ae*, while the RSD^r^ value ranged from 0.22% to 0.57% for the GHB119 assay, from 0.07% to 0.50% for the T304-40 assay and from 0.15% to 0.36% for the *Cry2Ae* assay (Table 4). The SD^R^ and RSD^R^ values for GHB119, T304-40 and *Cry2Ae* detection ranged from 0.01% to 0.15% and 0.02 to 0.46%, respectively (Table 4). The results of the repeatability test indicated that the quadruplex real-time PCR method is stable and repeatable for simultaneous quantitative detection of GHB119, T304-40 and *Cry2Ae*.

**Table 4:** **Repeatability of the quadruplex real-time PCR method for GM cotton GHB119, T304-40 and** ***Cry2Ae*** **detection**

**Amount of DNA (copies/reaction)**	**Ct value**					**SD**^r*^	**RSD**^**r**^**(%)**	**SD**^R*^	**RSD**^**R**^**(%)**
	**REP1**	**REP2**	**REP3**	**REP4**	**Mean**				
GHB119									
11600	24.39	24.51	24.57	24.40	24.47	0.09	0.36	0.05	0.19
2320	26.80	26.83	26.96	26.78	26.84	0.08	0.30	0.03	0.12
464	29.23	29.28	29.46	29.49	29.37	0.13	0.43	0.03	0.11
232	30.26	30.41	30.50	30.35	30.38	0.10	0.34	0.06	0.19
92.8	31.69	31.72	31.83	31.69	31.73	0.07	0.22	0.08	0.25
46.4	32.53	32.89	32.93	32.69	32.76	0.19	0.57	0.11	0.33
T304-40									
11600	24.68	24.70	24.72	24.69	24.70	0.02	0.07	0.05	0.20
2320	27.04	27.07	27.10	27.23	27.11	0.08	0.30	0.02	0.08
464	29.67	29.68	29.70	29.84	29.72	0.08	0.26	0.01	0.02
232	30.72	30.77	30.89	30.96	30.83	0.11	0.36	0.05	0.17
92.8	31.96	32.05	32.16	31.78	31.99	0.16	0.50	0.10	0.30
46.4	33.08	33.25	33.36	33.43	33.28	0.15	0.46	0.15	0.46
*Cry2Ae*									
11600	23.29	23.32	23.34	23.21	23.29	0.06	0.24	0.04	0.17
2320	25.64	25.68	25.71	25.73	25.69	0.04	0.15	0.04	0.17
464	28.03	28.15	28.17	28.22	28.14	0.08	0.28	0.08	0.27
232	29.02	29.19	29.20	29.24	29.16	0.10	0.33	0.05	0.17
92.8	30.63	30.68	30.83	30.85	30.75	0.11	0.36	0.07	0.23
46.4	31.99	32.02	32.05	32.14	32.05	0.06	0.20	0.10	0.31

*SD^r^: standard deviation of four parallel reactions; SD^R^: standard deviation of three experiments; RSD^r^: relative standard deviation of four parallel reactions; RSD^R^: relative standard deviation of three experiment.

### Blind sample analyses using the established quadruplex real-time PCR method

To verify the applicability of this quadruplex PCR method for quantification, six premixed samples (S1–S6) containing different percentages of GHB119 and T304-40 were prepared and analyzed to evaluate the accuracy and precision. The results showed that for quantification of GHB119, the mean values in the six blind samples were 0.48, 5.02, 2.47, 0.99, 0.00 and 0.47%, respectively (Table 5). The bias (between the experimental values and the given values) ranged from -6.03% to 0.31%. The SD value ranged from 0.01 to 0.12 and the RSD value ranged from 2.02 to 4.17%. Similarly, for T304-40 quantification, the mean values of six samples were 4.95, 0.93, 0.47, 2.51, 0.47 and 0.00%, respectively (Table 5). The bias ranged from -6.84% to 0.54%, the SD ranged from 0.02 to 0.13 and the RSD ranged from 1.82 to 13.95%. For *Cry2Ae* gene analysis, the mean GM contents of S1–S6 were found to be 0.51, 4.87, 2.67, 1.08, 0.00 and 0.53%, respectively. The bias values ranged from -0.03% to 0.17%, SD values ranged from 0.01 to 0.20 and RSD values from 1.14% to 4.31%. These values were all in the acceptable ranges [16].

**Table 5:** Quantitative analysis of six blind samples

**Sample code**	**Event or gene**	**GM content (%)**	**REP1 (%)**	**REP2 (%)**	**REP3 (%)**	**REP4 (%)**	**Mean value (%)**	**SD**	**RSD (%)**	**Bias (absolute value)**	**Bias (%)**
S1	GHB119	0.5	0.47	0.48	0.51	0.45	0.48	0.02	4.17	-0.02	-3.96
	T304-40	5.0	4.86	4.87	5.02	5.03	4.95	0.09	1.82	-0.05	-1.08
	*Cry2Ae*	0.5	0.49	0.50	0.50	0.53	0.51	0.02	3.79	0.01	1.22
S2	GHB119	5.0	4.94	4.99	4.94	5.19	5.02	0.12	2.39	0.02	0.31
	T304-40	1.0	1.02	1.07	0.84	0.80	0.93	0.13	13.95	-0.07	-6.84
	*Cry2Ae*	5.0	4.67	5.14	4.85	4.82	4.87	0.20	4.07	-0.13	-2.61
S3	GHB119	2.5	2.46	2.41	2.51	2.50	2.47	0.05	2.02	-0.03	-1.14
	T304-40	0.5	0.46	0.49	0.48	0.45	0.47	0.02	4.25	-0.03	-5.86
	*Cry2Ae*	2.5	2.72	2.66	2.64	2.67	2.67	0.03	1.14	0.17	6.93
S4	GHB119	1.0	0.95	1.03	1.02	0.95	0.99	0.04	4.06	-0.01	-1.36
	T304-40	2.5	2.46	2.54	2.48	2.56	2.51	0.05	1.99	0.01	0.54
	*Cry2Ae*	1.0	1.06	1.08	1.09	1.09	1.08	0.01	1.22	0.08	8.23
S5	GHB119	0.0	0.00	0.00	0.00	0.00	0.00	0.00	0.00	0.00	0.00
	T304-40	0.5	0.44	0.44	0.46	0.53	0.47	0.04	8.52	-0.03	-6.10
	*Cry2Ae*	0.0	0.00	0.00	0.00	0.00	0.00	0.00	0.00	0.00	0.00
S6	GHB119	0.5	0.47	0.46	0.47	0.48	0.47	0.01	2.13	-0.03	-6.03
	T304-40	0.0	0.00	0.00	0.00	0.00	0.00	0.00	0.00	0.00	0.00
	*Cry2Ae*	0.5	0.52	0.51	0.52	0.56	0.53	0.02	4.31	0.03	5.80

## Conclusions

To improve the implementation of the labeling policy, the supervision of unauthorized GM crop events in China was very important. In this study, a novel quadruplex real-time PCR method for simultaneous detection and quantification of GM cotton events GHB119 and T304-40 and Cry2Ae gene was developed. The low LODs, LOQs and the high accuracy and precision in quantification indicated that the developed quadruplex real-time PCR were suitable for the qualitative and quantitative detections of GHB119, T304-40 and Cry2Ae in cotton or cotton-derived products.

The accuracy and precision of the quantification for blind sample using our quadruplex quantitative PCR method are similar to those of many other quantitative analysis methods for GM soybean and maize [3,17]. Therefore, the quadruplex real-time PCR method developed in this study is reliable and suitable for the quantitative detection of GHB119, T304-40 and *Cry2Ae* in cotton or cotton-derived products.

## Abbreviations

BHQ: The Black Hole Quencher; Ct: Cycle threshold; Cy5: Cyanine dye 5; FAM: 6-carboyfluorescein; HEX: 6-carboxy-2′, 4, 4′, 5′, 7, 7′-hexachlorofluorescein; LNA: Locked nucleic acid; LOD: Limit of detection; LOQ: Limit of quantification; PCR: Polymerase chain reaction; qPCR: Quantitative polymerase chain reaction; ROX: 5-carboxy-x-rhodamine; RSD: Relative standard deviation; SD: Standard deviation; SNP: Single nucleotide polymorphisms; Tm: Melting temperature.

## Competing interests

The authors declare that they have no competing interests.

## Authors’ contributions

LX conceived of the study, participated in its design and coordination and performed the development of the quadruplex real-time PCR detection method. WX participated in the specificity and sensitivity tests of the quadruplex real-time PCR method. YJ participated in the blind samples analysis. LY participated in the screening of the primers and probes. HY participated in the design of the study and in drafting the manuscript. PL carried out the sequence alignments of *Cry* genes and the developed primers and probes. All authors read and approved the final manuscript.
